# Clinical and Radiologic Outcomes of Combined Use of Biocomposite and PEEK Suture Anchors during Arthroscopic Rotator Cuff Repair: A Prospective Observational Study

**DOI:** 10.3390/jcm9082545

**Published:** 2020-08-06

**Authors:** Jae-Hoo Lee, Yong-Beom Lee

**Affiliations:** Department of Orthopaedic Surgery, Hallym University Sacred Heart Hospital, Medical College of Hallym University, Chuncheon 24257, Korea; holleewho@gmail.com

**Keywords:** perianchor cyst, biocomposite anchor, PEEK anchor, arthroscopic rotator cuff repair

## Abstract

The aim of the current study was to evaluate the functional and radiologic outcomes of biocompatible non-absorbable PEEK (polyetheretherketone) and biocomposite (poly-L-lactic acid/poly(lactic-co-glycolic acid) 70% + β-tricalcium phosphate) anchors, especially in terms of perianchor cyst formation during the first six months postoperatively. We prospectively analysed 29 patients who underwent arthroscopic rotator cuff repair between March and May 2019. Both PEEK and biocomposite suture anchors were used as lateral anchors in one body. Clinical outcomes were assessed using the shoulder range of motion (ROM), visual analogue scale (VAS) for pain and satisfactory score, American Shoulder and Elbow Surgeons (ASES) score, and Simple Shoulder Test (SST). All these were obtained in patients preoperatively at 3 and 6 months after surgery. The imaging evaluation included perianchor cyst formation, anchor absorption, repaired cuff integrity, and retear pattern. All functional outcomes significantly improved over time. The biocomposite anchor had a statistically significant tendency to form higher grades of fluid collection at 3 months after surgery. However, the perianchor cyst reduced by the sixth postoperative month. Six months postoperatively, the functional outcomes were improved after rotator cuff repair and similar degrees of perianchor cyst formation were observed, regardless of the suture anchor material used.

## 1. Introduction

A rotator cuff tear is one of the most common pathologic conditions of the shoulder joint which orthopaedic surgeons frequently encounter. Suture anchors are commonly used for successful rotator cuff repair. The metallic anchor was the first to be introduced; however, complications, such as metallic artifacts which interfered with postoperative assessments on magnetic resonance imaging (MRI), were reported. Another complication was implant loosening and migration into a joint space which led to arthritis [1,2,3,4,5]. On the other hand, the earlier developed bioabsorbable anchors also yielded several anchor-related complications including osteolysis, synovitis, perianchor cyst formation, systemic allergic reaction, and foreign-body reactions [6,7,8,9]. To overcome these problems, the biocompatible, non-absorbable PEEK (polyetheretherketone) anchor and the biocomposite, bioabsorbable (poly-L-lactic acid/poly(lactic-co-glycolic acid) 70% + β-tricalcium phosphate; PLLA/PGA plus β-TCP) anchors were introduced.

The biocompatible PEEK anchor was widely used due to its strength resembling that of the human bone and the ease in its manufacturing and moulding. However, due to its hydrophobic and unabsorbable properties, PEEK had low bone integration and remained in the body. Thus, it had to be removed for revisional surgery. It was not feasible to choose the PEEK anchor in massive rotator cuff repairs due to high retear rates, which led to revisional surgery [10,11].

On the other hand, a newly introduced biocomposite, bioabsorbable anchor composed of 70% PLLA/PGA and 30% β-TCP yielded both properties of absorbability and catalysis of bony ingrowth [12,13]. Several studies have reported how the perianchor cyst formation negatively affected the maintenance of the anchors. However, none of these studies analysed cyst formation and its effect on cuff integrity at a very early period of rotator cuff healing because healing of the rotator cuff was achieved in the first six months.

The aim of the current study was to test the hypothesis that the biocomposite absorbable anchor was comparable to the non-absorbable biocompatible anchor in terms of perianchor cyst formation. We also aimed to assess whether the rotator cuff maintained its integrity and clinical outcomes even though a perianchor cyst had formed around the anchor during the first six months of rotator cuff healing.

## 2. Materials and Methods

### 2.1. Patient Selection

This study’s protocol was approved by the Institutional Review Board of Hallym University Sacred-Heart Hostpital (No. HALLYM2019-05-001). The randomized controlled trial was also registered (PRE20200727-001). Informed consent was obtained from all patients. We prospectively enrolled patients between March and May 2019. The inclusion criteria were as follows: (1) full-thickness rotator cuff tear verified by preoperative magnetic resonance imaging (MRI) or partial-thickness rotator cuff tear refractory to conservative treatment over 6 months, (2) arthroscopic suture bridge repair using both PEEK (4.75 mm, CrossFT™ knotless anchor; ConMed, Utica, NY, USA) and biocomposite anchor (70% PLGA/30% β-TCP, 4.75 mm Fix2Lock™; Kinex Osteonic, Seoul, South Korea) as a lateral row anchor, (3) availability for MRI evaluation to be taken preoperatively and at 3 and 6 months postoperatively to assess the rotator cuff integrity and status of the anchors, and (4) consent to join the study.

The exclusion criteria were as follows: (1) history of previous surgical treatment on the affected side wrist, elbow, or shoulder; (2) infectious, autoimmune, or systemic skeletal disease; (3) rotator cuff arthropathy or moderate to severe osteoarthritis or rheumatoid arthritis (*n* = 4); (4) unavailability for taking MRI due to implants such as pacemakers; (5) no consent to take part in the study; and (6) lost to follow-up before the second MRI could be taken at 6 months (*n* = 6).

Even though full-thickness tear was verified by preoperative MRI, if the torn rotator cuff was not retracted proximally beyond the articular surface of the humeral head and the patient manifested mild symptoms and had good function, then we recommended a conservative treatment.

Variable demographic factors can affect the healing course of a repaired rotator cuff. Maintenance of the inserted anchor is dependent on sex, age, hand dominancy, history of trauma, occupation, status of physical activity, smoking, diabetes mellitus, osteoporosis, thyroid disease, and previous history of steroid injections. These factors were collected prospectively and evaluated.

### 2.2. Sample Size Calculation

The incidences of perianchor cyst formation using non-absorbable and bio-absorbable anchors in rotator cuff repair were 10.6% and 44.6%, respectively [14]. By power analysis, a sample size of 26 patients in each group was required for a power of 80% with a type I error of 0.05. A characteristic point in this study was that the comparison between groups was conducted within the same individual. Twenty-eight patients were consequently enrolled in the study.

### 2.3. Surgical Procedure and Rehabilitation

All surgical procedures were conducted by a single surgeon (Yong-Beom Lee) with over 20 years of experience in a single institution. The size of the rotator cuff tear was measured using a probe under arthroscopic view. Biceps tenotomies (*n* = 9; 31% of total patients) were performed if the long head of the biceps tendon was dislocated or torn. Acromioplasties were also performed to decompress the bony spurs in all cases.

**Figure 1:**
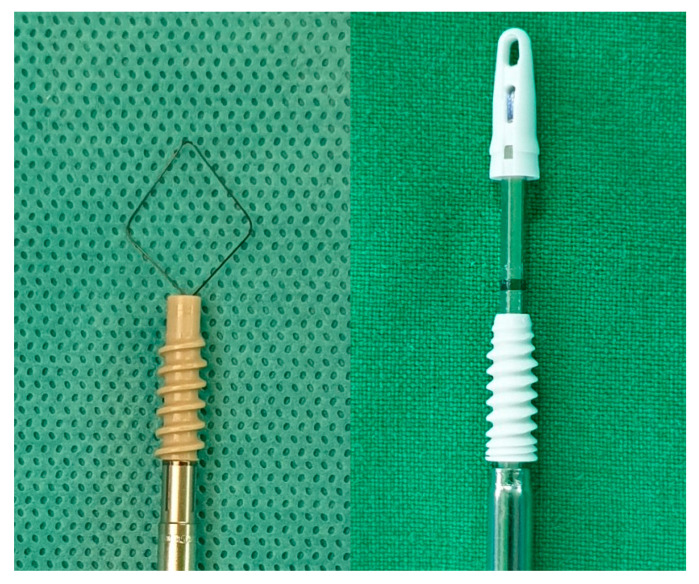
The anchors utilized in the current study have the same 4.75 mm diameters. The 4.75 mm CrossFT™ knotless anchor (left: ConMed, Utica, NY, USA) and 4.75 mm Fix2Lock™ anchor (right: Kinex Osteonic, Seoul, Korea) are shown.

The footprint of the tear was prepared using a motorized bur to eliminate soft tissue remnants and facilitate bone-tendon healing. The two medial row anchors were inserted at the medial border of the footprint of greater tuberosity adjacent to the articular surface and the lateral anchors were placed on the lateral aspect of humerus in regard to the shape of bridge figuration. The repairs were done using the suture bridge technique introduced by Park et al. [15]. The bridge technique used two medial row knot ties with Y-knot^®^ RC all-suture anchor system (ConMed, Utica, NY, USA). For lateral row fixation, two type of anchors were used: a 4.75 mm Fix2Lock™ (Kinex Osteonic, Seoul, Korea) and a 4.75 mm CrossFT™ knotless anchor (ConMed, Utica, NY, USA) (Figure 1). The decisions regarding the placement of lateral anchors either anteriorly or posteriorly were made according to a pre-set randomization table. The patients received rehabilitation after surgery. One week after surgery, the pendulum exercise was started. An active-assisted range of motion (ROM) exercise was started at 6 weeks postoperatively. After 3 months postoperatively, the patients were permitted to start isometric muscle exercises with a rubber band. Minor sport activities were permitted after full ROM and adequate muscle strength had been recovered [16].

### 2.4. Clinical Outcome

For assessment of clinical outcomes, the shoulder ROM, visual analogue scale (VAS) for pain and satisfactory score, American Shoulder and Elbow Surgeons (ASES) score, and Simple Shoulder Test (SST) were obtained in all patients preoperatively and at 3 and 6 months after surgery. The functional scores were measured by a research assistant (HNY) at every visit. A goniometer was used to evaluate the range of motion (ROM) of forward flexion, external rotation, and internal rotation. Internal rotation was assessed as the highest vertebral level that the tip of the thumb could reach. The levels of the vertebrae were numbered from below the sacrum (0) to the fourth thoracic vertebra (15) in series. ROM was measured by the same research assistant (Ha-Na Yoo). The VAS, ranging from 0 to 10, was used for subjective pain assessment.

### 2.5. Postoperative Imaging Evaluation

All patients underwent postoperative MRI twice during the third and sixth months after surgery (mean ± standard deviation (SD); 3.58 ± 0.97 (1.18–5.54 months) and 6.52 ± 1.36 (4.26–9.54 months), respectively). All shoulder MRI evaluations were performed using 3.0 T (Signa HDx; GE healthcare, Milwaukee, WI, USA) with a dedicated shoulder coil. Patients were placed in the supine position with the forearm in a semi-pronated (neutral) position. The following MRI protocols were used: axial fast spin-echo proton-density weighted image with fat saturation (repetition time (TR)/echo time (TE): 2300–3900/30–60 ms, slice thickness: 3 mm, interslice gap: 0 mm, field of view (FOV): 16 cm); oblique coronal fast spin-echo T2-weighted images with fat saturation (TR/TE: 2300–4600/30–50 ms, slice thickness: 2 mm, interslice gap: 0 mm, FOV: 16 cm); and sagittal fast spin-echo T2-weighted images with fat saturation (TR/TE: 2300–4600/30–50 ms, slice thickness: 3 mm, interslice gap: 0.3 mm, FOV: 16 cm).

Perianchor cyst formation was graded on coronal and axial MRI scans according to the classification introduced by Kim et al. [14]: grade 1, linear fluid collection around anchor; grade 2, local collection of fluid around any location of the anchor; grade 3, fluid collection around the entire length of the anchor with the cyst diameter less than twice the anchor diameter; and grade 4, cyst diameter larger than grade 3. Anchor absorption was graded on coronal and axial MRI scans according to the classification of Haneveld et al. [17]. Grade 1 indicated a clearly visible structure, grade 2 indicated a visible structure, grade 3 indicated a partially visible structure, and grade 4 described a structure which could not be delineated from the surrounding tissue. Tendon integrity was classified into five categories according to Sugaya’s classification using T2-weighted oblique coronal MRI scans [18]. Type I indicated a repaired cuff that had sufficient thickness with homogeneously low intensity on each image. Type II indicated sufficient thickness associated with a partial high-intensity area, while type III had insufficient thickness without discontinuity. Type IV showed the presence of a minor discontinuity in more than one slice of each image, suggesting a small tear. Type V indicated the presence of a major discontinuity on each image, suggesting a medium or large tear [19]. Retear pattern was categorized according to Rhee’s classification [20] on T2-weighted oblique sagittal scans: type 1, the cuff tissue repaired at the rotator cuff’s insertion site was not at all observed to be remaining on the greater tuberosity and type 2, remnant cuff tissue remained at the insertion site despite retear.

Fatty degeneration was evaluated for a torn muscle with the 5-stage grading system introduced by Goutallier et al. [21,22] on T1-weighted MRI scans. The size and extent of the tear were measured on oblique coronal and oblique sagittal MRI views since these increased the failure rate of a repaired rotator cuff. [23] All the MRI findings were evaluated by the two authors. When there were disagreements, we determined the respective grades through discussions.

### 2.6. Statistical Analysis

All statistical analyses were conducted with the SPSS (version 26; IBM SPSS, Chicago, IL, USA) software. Functional assessments over time were analysed with repeated measures of 1-way analysis of variance (RM-ANOVA), paired T-test, and Wilcoxon signed rank test. The χ^2^ test and Fisher’s exact test were used for categorical variables. The statistical significance was set at *p* < 0.05.

## 3. Results

### 3.1. Comparison between Biocomposite and PEEK Anchors

#### 3.1.1. Patients’ Demographics

The patients’ demographics are summarized in Table 1.

#### 3.1.2. Functional Assessment

All functional outcomes significantly improved over time (Table 2). However, the patients could not reach the “no pain” status according to the functional scores at 6 months. The mean ROM either showed the least improvement or remained the same. No statistical significance was observed, except for external rotation.

#### 3.1.3. Radiographic Assessment

Among 29 patients, 24 (92.8%) had full-thickness rotator cuff tear, while 16 (55.2%) patients had only single supraspinatus tears. On the preoperative MRI findings, even distributions of fatty infiltrations according to the Goutallier’s classification were observed (Table 3).

#### 3.1.4. Perianchor Cyst Formation

**Figure 2:**
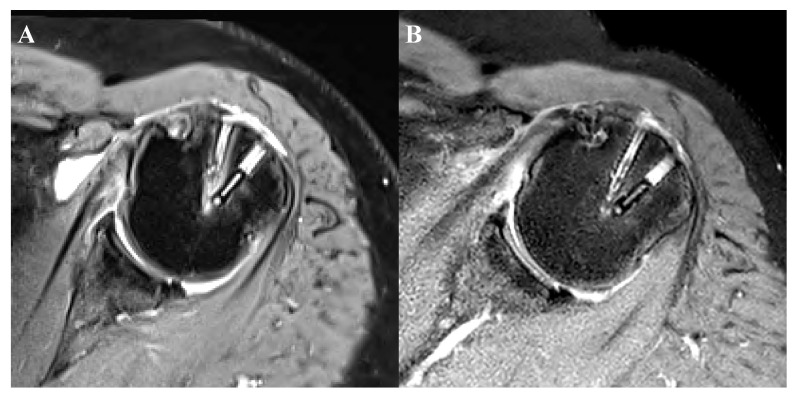
A 74-year-old woman with full-thickness supraspinatus tear underwent arthroscopic rotator cuff repair. Three months after the surgery, grade 3 fluid collection was seen forming around a biocomposite anchor (**A**). Subsequently, the fluid was noted to have reduced to grade 1 during the sixth postoperative month (**B**).

Periachor cyst formation was observed around both biocomposite and PEEK anchors. During the early period (until 3 months) of rotator cuff healing, cyst formation showed similar compositions according to Kim’s classification [14]. However, at 6 months, majority of the cyst formation around the PEEK anchor belonged under grade 2. On the other hand, the biocomposite anchor showed an opposite distribution. Perianchor cyst formation was shown in 72.4% and 58.6% of patients using biocomposite and PEEK anchors at the third postoperative month, respectively. Subsequently, these rates increased to statistically significant values of 75.9% and 72.4% at 6 months, respectively. Comparing the two anchors subdivided as low (0, 1) and high grade (2, 3, and 4), the PEEK anchor had significantly better outcomes in terms of fluid formation during the third postoperative month. Otherwise, no significant difference was observed during the sixth postoperative month (Figure 2A,B). None of the anchors were absorbed during the first six postoperative months. The retear rate was noted in 4 (13.8%) patients who had a mean age of 61 years (range, 54–74 years). The mean tear size was 34 mm (ranged, 20–50 mm). Type II retear pattern was observed in all four retear cases (Table 4). There was no statistical significance of the formation of perianchor cyst according to the tear size by using the χ^2^ test and Fisher’s exact test (biocomposite anchor at 3 months, *p* = 0.798; at 6 months, *p* = 0.623; PEEK anchor at 3 months, *p* = 0.810; at 6 months, *p* = 0.944).

## 4. Discussion

In this study, we evaluated perianchor cyst formation around two distinct anchors and its effects on the patients’ clinical and radiologic outcomes. The most substantial finding in the current study was that the biocomposite anchor formed comparable amounts of fluid collection with the PEEK anchor. Even though the grade of perianchor cyst formation around the biocomposite anchor induced more fluid collection during the early period of rotator cuff healing, the perianchor cyst faded over time and did not negatively affect both the integrity of repaired rotator cuff and the functional outcomes of patients during the first six months.

Perianchor cyst formation was observed in 72.4% and 58.6% of patients using biocomposite and PEEK anchors during the third postoperative month, respectively. These rates increased to 75.9% and 72.4%, respectively, at 6 months. Kim et al. reported that 7.9–50% of two subgrouped participants using three distinct biocomposite biodegradable suture anchors had fluid collection around the anchors approximately one year postoperatively (12.7 ± 0.9 months and 12.6 ± 1.8 months, respectively). However, the rates of severe perianchor cyst formation were reported 0 to 2.6%. These findings are comparable to the results of the current study, in which the rate of the perianchor cyst formation was 0% in both groups at 6 months [24]. Chung et al. reported that perianchor cyst formation was shown in 60.3% of patients using biocomposite anchors approximately 7 months after operation [25]. Ro et al. reported that suture, bioabsorbable, and PEEK anchors showed perianchor cyst occurrence rates of 29.9%, 36.1%, and 27.5% respectively at 9.6 months postoperatively [26]. In the present study, although there was a relatively high perianchor cyst occurrence rate, the majority of the perianchor cysts had low-grade fluid collections. We can surmise that fluid collection increases during the early period due to anchor micromotion from the tensile load of the repaired rotator cuff. This may decrease with time depending on bony ingrowth, repair site stability, and fluid resorption.

In the current study, we used a domestically produced, biocomposite, bioabsorbable suture anchor—Fix2Lock™ (Kinex Osteonic, Seoul, Korea). This anchor is composed of 70% PLGA and 30% β-TCP. It showed comparable outcomes with the PEEK anchor in terms of perianchor fluid collection. Chung et al. reported that the biocomposite bioabsorbable anchor (*n* = 38, Fixone anchor B; Aju Medical, Seoul, South Korea) was not inferior to the Healix BR™ anchor (*n* = 40; Depuy Mitek, Raynham, MA, USA) when used as medial row anchors (both 30% β-TCP and 70% PLGA). These were comparable in terms of failed healing rate (13.2% vs. 15.0%), perianchor cyst formation (60.5% vs. 60.0%), and major anchor-related complications evaluated by radiology at 6.3 ± 0.9 and 7.8 ± 1.2 postoperative months, respectively. In the follow-up study, the formation of perianchor cyst decreased with time and was observed only in 7 patients (18.4%) at 18 months after surgery [25,27] On the other hand, Shin et al. reported that the bony ingrowth into PEEK anchors occurred two times faster than biocomposite anchors [28]. The PEEK anchor used in the study was newly developed and open-vented to facilitate bony ingrowth. Nevertheless, it had the disadvantage of remaining inside the body and interfered with postoperative radiographic evaluations and subsequent revision surgeries with occupying bone stock.

The strength of the current study is that it was designed and conducted prospectively. Hence, we obtained quality data and had no lack of MRI evaluations at every visit. Second, the anchors were used for lateral, not medial row, fixation. Therefore, we could control the confounding factor of pulling-out tensile stress on the anchors. Third, we inserted two distinct anchors in one patient; hence, we were able to control the environmental factors that may have influence on the anchor maintenance. Similar to an identical twin study, this also eliminated selection bias from patient-based factors and led to improved study liability. Fourth, we controlled the size and geometry of the anchor thread and pitch. Therefore, the 4.75 mm CrossFT™ knotless anchor was selected as the control anchor due to its similar thread and pitch geometry with the Fix2Lock™ anchor.

This study had several limitations. This study focused on the first six months when the healing of the repaired rotator cuff would be achieved. We do not have long term follow-up data on how the perianchor cysts changed. A follow-up study is needed to document retear occurrence and assess when the bioabsorbable anchor would be completely absorbed. However, this may be estimated based on previously published studies [27,29]. Second, applying two distinct anchors into one body as the strong point of this study simultaneously acted as an obstacle in assessing the functional outcomes. Further studies are warranted using only the PEEK or biocomposite bioabsorbable anchor as the control group. Third, we graded the perianchor cysts according to Kim’s classification [14]. Both the authors determined the grades accordingly through discussions when some disagreements were met. However, there may be grading bias because of its subjectivity. A third observer who is not familiar with the study should be included for radiologic assessment in further studies.

## 5. Conclusions

Functional outcomes were improved after rotator cuff repair. Similar degrees of perianchor cyst formation were observed at 6 months postoperatively, regardless of the suture anchor material used. However, we found that the perianchor cyst was formed in relatively large amounts using biocomposite anchor during the first three months postoperatively. Nevertheless, this did not have influence on the postoperative rotator cuff integrity. The fluid reduced with time by the sixth postoperative month.

## Figures and Tables

**Table 1:** Patients’ demographics.

	29 Patients in Total
Gender (male–female (%))	11 (37.9):18 (62.1)
Age (mean (range, SD))	57.8 (27–74, 9.0)
Involvement of dominant extremity (%)	20 (69%)
History of trauma (%)	10 (34.5%)
Occupation (%)	
Manual labourer	3 (10.3)
Indoor office worker	10 (34.5)
Unemployed	10 (34.5)
Others	6 (20.7)
Regular physical activity (%)	6 (20.7)
Smoking	4 (13.8)
Diabetes mellitus	7 (24.1)
Osteoporosis (*n* (%), mean bone mineral density)	5 (17.2%), −3.16
Thyroid disease	5 (17.2)
Previous history of steroid injection	12 (41.4)

SD, standard deviation.

**Table 2:** Functional assessment.

	Preoperative	Postoperative at 3 Months	Postoperative at 6 Months	*p*-Value
	Mean (Range, SD)			
pVAS	5.6 (1–10, 2.1)	3.2 (0–6, 1.5)	2.6 (1–5, 0.9)	0.001 *
SST	4.7 (0–10, 2.7)	6.4 (3–9, 1.7)	7.5 (4–9, 1.6)	0.005 *
UCLA	17.1 (3–34, 6.6)	21.6 (10–30, 5.7)	24 (15–30, 5.1)	0.001 *
ASES	53.6 (11.9–90, 20.7)	72.5 (49.1–89.1, 11.7)	79.1 (59.1–90, 7.3)	<0.001 *
CSS	45.6 (14–82,18.5)	54.8 (25–79, 14.3)	60.7(35–79, 12.1)	0.004 *
Range of motion				
Forward extension	154.31 (0–180, 41.1)	165.4 (100–180, 27)	173.3 (90–180, 21.4)	0.137
External rotation	63.3 (10–90, 23.5)	75.4 (40–90, 14.8)	85 (60–90, 9.2)	0.001 *
Internal rotation	7 (T11, 1–15, 5.9)	2.32 (L3–4, 1–15, 2.9)	6.4 (T11–12, 1–15, 6.4)	0.752

* Statistical significance; SD, standard deviation; pVAS, pain visual analog scale; SST, Simple Shoulder Test; UCLA, the rating scale of the University of California at Los Angeles; ASES, the shoulder index of the American Shoulder and Elbow Surgeons; CSS, constant shoulder score. For measuring the internal rotation, the level of the vertebra was numbered serially from 0 point (below L5) to 15 points (at T4 level).

**Table 3:** Radiographic assessment.

	Total 29 Patients
Tear pattern (*n*, (%))	
Full thickness	24 (82.8)
Partial thickness-bursal	3 (10.3)
Partial thickness-articular	2 (6.9)
Muscle Involvement	
Supraspinatus only	16 (55.2)
Supraspinatus and infraspinatus	4 (13.8)
Surpraspinatus + subscapularis	5 (17.2)
Supraspinatus and infraspinatus + subscapularis	4 (13.8)
Tear size of supraspinatus (mean, (range, SD))	18.7 (5–50, 11.8)
Degree of fatty infiltration (grade, (%))	
Grade 0	1 (3.4)
Grade 1	8 (27.6)
Grade 2	10 (34.5)
Grade 3	4 (13.8)
Grade 4	6 (20.7)
Biceps lesion (*n*, (%))	9 (31)
Capsulitis	3 (10.3)

*n*, number; SD, standard deviation.

**Table 4:** Results of perianchor cyst formation.

Anchor Placement (Biocomposite/PEEK), *n* (%)						
Anterior	21 (72.4)/8 (27.6)					
Posterior	8 (27.6)/21 (72.4)					
Cyst formation, *n* (%)	3 months		*p*-value	6 months		*p*-value
	Biocomposite	PEEK		Biocomposite	PEEK	
Grade 0	8 (27.6)	12 (41.4)	0.085	7 (24.1)	8 (27.6)	0.581
Grade 1	7 (24.1)	11 (37.9)		11 (37.9)	11 (37.9)	
Grade 2	6 (20.7)	1 (3.4)		5 (17.2)	6 (20.7)	
Grade 3	5 (17.2)	4 (13.8)		3 (10.3)	4 (13.8)	
Grade 4	3 (10.3)	1 (3.4)		3 (10.3)	-	
Low grade (grade 0 or 1)	15 (51.7)	23 (79.3)	0.028 *	18 (62.1)	19 (65.5)	0.787
High grade (grade 2, 3, or 4)	14 (48.3)	6 (20.7)		11 (37.9)	10 (34.5)	
Anchor absorption	none	none		none	none	
Retear, *n* (%)	4 (13.8)			4 (13.8)		
Retear pattern (type I/II)	0/4			0/4		

* Statistical significance; *n*, number. Biocomposite represents the biocomposite bioabsorbable anchor; PEEK represents the polyetheretherketone anchor.
